# Fibromuscular Dysplasia Clinical Phenotype Manifesting as a Distal Spontaneous Coronary Artery Dissection in a Middle-Aged Man

**DOI:** 10.7759/cureus.78127

**Published:** 2025-01-28

**Authors:** Jonathan Van Name

**Affiliations:** 1 Internal Medicine, University of Florida College of Medicine, Gainesville, USA

**Keywords:** connective tissue disease, fmd and scad, migraine, pulsatile tinnitus, spontaneous coronary dissection

## Abstract

Fibromuscular dysplasia (FMD) is a non-atherosclerotic, non-inflammatory vascular disease of medium-sized arteries that causes abnormal cellular growth in arterial walls and most commonly affects young to middle-aged women (20-50 years of age). While FMD often involves the renal arteries, it can affect any arterial bed. FMD has a characteristic angiographic appearance of a “string of beads." However, rarely patients may present with an FMD-like clinical phenotype without characteristic angiographic FMD evidence. An FMD phenotype consists of a specific constellation of arterially-induced symptomatology (e.g., pulsatile tinnitus in cerebrovascular FMD) in the setting of acute arterial disturbances typically found in FMD (e.g., arterial dissection). This case report discusses a cerebrovascular FMD clinical phenotype manifesting as a distal spontaneous coronary artery dissection (SCAD) in a middle-aged man with chronic migraines, pulsatile tinnitus, and no prior cardiac history. In this case, a patient presented to the emergency room with elevated high-sensitivity troponin levels and anginal chest pain thought to be secondary to a non-ST-elevated myocardial infarction (NSTEMI). A left heart catheterization revealed non-obstructive atherosclerosis and a spontaneous coronary artery dissection in the distribution of the distal left anterior descending artery. To assess potentially involved arterial beds, further work-up consisting of a bilateral carotid and renal artery duplex demonstrated significant carotid arterial tortuosity, a finding consistent with cerebrovascular FMD. Per consultation with neurology and cardiovascular surgical services, a computed tomography angiography (CTA) aorta and CTA head and neck were obtained, which demonstrated subtle irregularities and tortuosities concerning for FMD but without characteristic findings. Further medical optimization was initiated for blood pressure and migraine symptomatology control. After completion of the diagnostic FMD work-up, the patient was discharged with monthly cardiovascular and neurological follow-up. This case report illustrates the important multifactorial practice of history-taking, laboratory evidence evaluation, and diagnostic imaging interpretation to ascertain the correct diagnosis in an atypical presentation of an uncommon disease spectrum.

## Introduction

Fibromuscular dysplasia (FMD) is a rare, vascular disease of medium-sized arteries of unknown etiology that can lead to arterial stenosis, vascular dissection, aneurysmal formation, and arterial tortuosity. While most instances of FMD involve the renal arteries, there may also be involvement of the carotids, vertebral arteries, and less commonly the external iliac arteries [1]. FMD most commonly presents in young to middle-aged women but can affect any age and sex [2]. Although the diagnosis of FMD involves distinctive angiographic findings of alternating luminal bulging and narrowing, FMD-like arteriopathies exist along a spectrum presenting with variable clinical phenotypes [3]. The clinical manifestations of FMD are largely dependent on the affected vascular territory [1]. In cerebrovascular FMD, for instance, patients typically present with recurrent and intractable migraine-like headaches, pulsatile tinnitus, dizziness, transient ischemic attack (TIA)/stroke, and Horner syndrome. However, in patients with arterial FMD, patients may experience flank pain, persistent hypertension, and abdominal pulsations. While most of these symptoms are non-specific, this report demonstrates that a specific constellation of symptoms in the setting of acute vascular disturbance (e.g., arterial dissection, highly tortuous arteries, etc.) indicates an arteriopathy with an FMD-like clinical phenotype even if classic FMD angiographic features are not present.

Multiple variants of FMD exist based on the distribution of collagenous deposition in the arterial wall, and they are not mutually exclusive of one another: medial FMD, intimal FMD, and adventitial FMD. Conventionally, FMD is diagnosed angiographically, with the “string of beads” being the most common appearance, indicating alternating areas of stenosis and dilations in the arterial wall. However, some patients may possess certain angiographic features of FMD (e.g., arterial tortuosity, dissection, subtle arterial wall irregularities) and similar FMD symptoms without the classic "string of beads" appearance. Patients in this category should undergo further evaluation for other similarly presenting connective tissue disorders. Specifically, two prior case reports demonstrate that patients with vascular Ehlers-Danlos syndrome often present with a similar FMD clinical phenotype given the vascular collagen integrity deficit caused classically by collagen genetic mutations [4,5]. Therefore, imaging and a thorough evaluation of a patient’s past medical, family, and genetic histories (if available) are critically important in the evaluation of diseases along the suspected connective tissue disorder spectrum to help distinguish underlying etiologies.

As aforementioned, arteriopathies with an FMD clinical phenotype may present as arterial dissection, tortuosity, or aneurysmal formation, particularly in the setting of medial hyperplasia. Further, the characteristic “string of beads” angiographic feature rarely occurs in coronary arteries despite a cohort study demonstrating that FMD was found in 25-86% of spontaneous coronary artery dissection (SCAD) cases [6,7]. This finding suggests that FMD with coronary involvement has an atypical form compared to other arterial beds; though these studies have a broad range due to differences in diagnostic criteria. While FMD primarily affects women, the development of SCAD is a concerning finding that warrants further FMD evaluation regardless of other patient characteristics. Additionally, patients with unexplained aortic aneurysms and highly tortuous arterial beds should also undergo further FMD and subsequent genetic evaluation. This case report discusses a patient with symptomatology and arterial disturbances consistent with FMD, despite lacking some of the characteristic angiographic FMD features. In this case, this patient's SCAD is attributed to an FMD phenotype given the constellation of the patient's symptoms (i.e., pulsatile tinnitus, chronic migraines) in the setting of tortuous carotid arteries. Additionally, this report highlights the improvement of FMD-induced symptoms (e.g., migraine headaches) when tailored therapy is provided. The atypical presentation of FMD in this case report emphasizes the importance of symptom evaluation and medical history, augmenting the current literature on this topic.

## Case presentation

**Figure 1:**
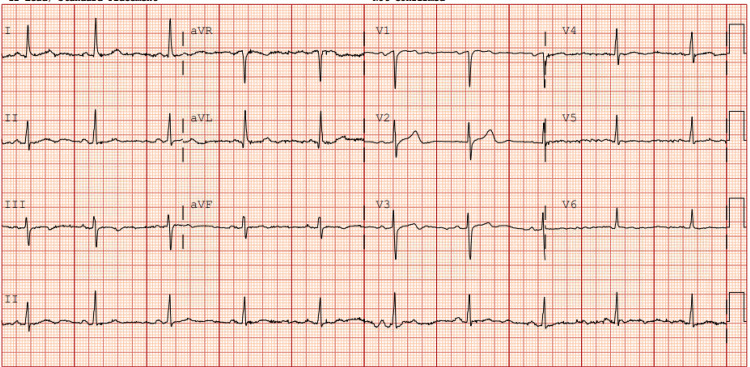
Initial 12-lead EKG on admission. The 12-lead EKG on admission shows normal sinus rhythm with heart rates at a baseline of 55-60 bpm. This EKG was obtained during the patient's cardiac troponin elevation but showed no evidence of any ST or other ischemic changes.

A 54-year-old African American male individual in the United States with a history of chronic migraines non-responsive to triptan therapy presented to the hospital with a three-day history of intermittent chest pain described as a “dull ache that started in center of chest and radiated from bottom of neck down left arm.” Upon interviewing the patient, the patient denied any genetic conditions or family history of cardiovascular or connective tissue disorders. In the emergency room, the patient was found to be hemodynamically stable, with vitals: blood pressure (BP) 149/119 mmHg, heart rate (HR) 56 bpm, respiratory rate (RR) 15 breaths/min, temperature 36.7°C, O2 100% saturation. Given his intermittent chest pain, the patient received a 12-lead electrocardiogram (EKG) which showed normal sinus rhythm with no ischemic or dynamic EKG changes, suggesting that a transmural infarct was less likely (Figure 1). Initial high-sensitivity cardiac troponin levels were 21 ng/L, but increased to 2,066 ng/L and 15,184 ng/L at two-hour and six-hour checks, respectively. Furthermore, inflammatory markers were negative with an erythrocyte sedimentation rate (ESR) of 6 MM/HR and C-reactive protein (CRP) of 0.51 mg/L. A chest X-ray at the time of presentation was unremarkable with no cardiopulmonary process.

**Figure 2:**
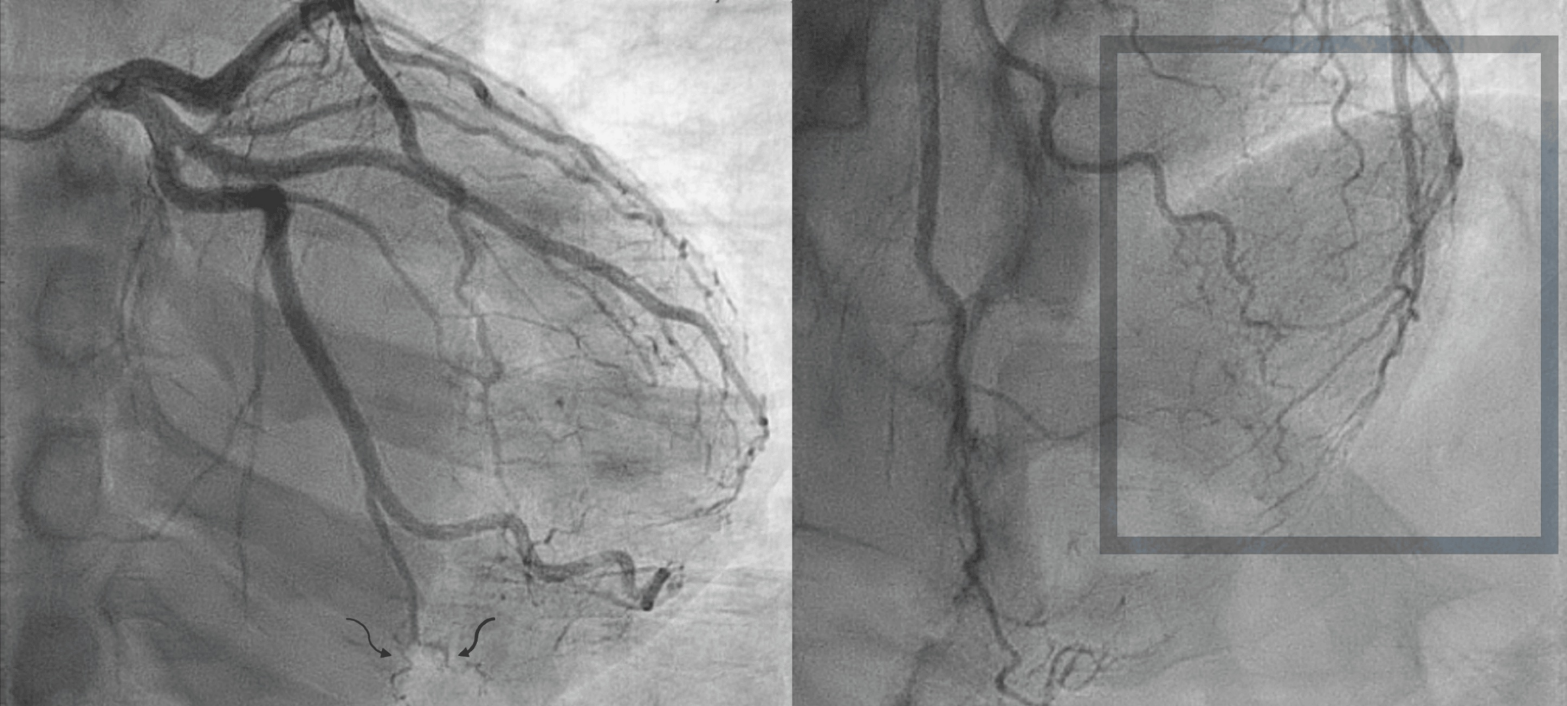
Angiographic films demonstrating distal spontaneous coronary artery dissection. Left: Curvy black arrows pointing to the distal left circumflex demonstrating coronary tortuosity. Right: Gray box in the distal left anterior descending distribution indicating high degree of coronary tortuosity and spontaneous coronary artery dissection.

The patient was started on a nitroglycerin drip for his chest pain and admitted to the in-patient cardiology service for high-risk chest pain secondary to a presumed non-ST elevated myocardial infarction (NSTEMI). After controlling the patient’s chest pain, further past medical history was obtained. The patient's past medical history is notable for well-controlled hypertension; social history was positive for a 20-pack-year smoking history with no tobacco use in the past 15 years. The patient reported a 15-year extensive history of migraine headaches that were refractory to triptan and prophylactic therapy, with no family history of headache disorder or other notable conditions. He additionally endorsed “hearing swishing sounds in ears,” which was described in a way consistent with pulsatile tinnitus. Further, the patient described experiencing transient neurological weakness consistent with a transient ischemic attack nearly two years prior. Given the aforementioned troponin elevation, the patient was taken for an urgent left heart catheterization (LHC), which demonstrated mild non-obstructive coronary disease but a distal spontaneous coronary artery dissection in the distribution of the left anterior descending artery (Figure 2). Revascularization was not performed, and risk modification measures were aggressively attempted.

**Figure 3:**
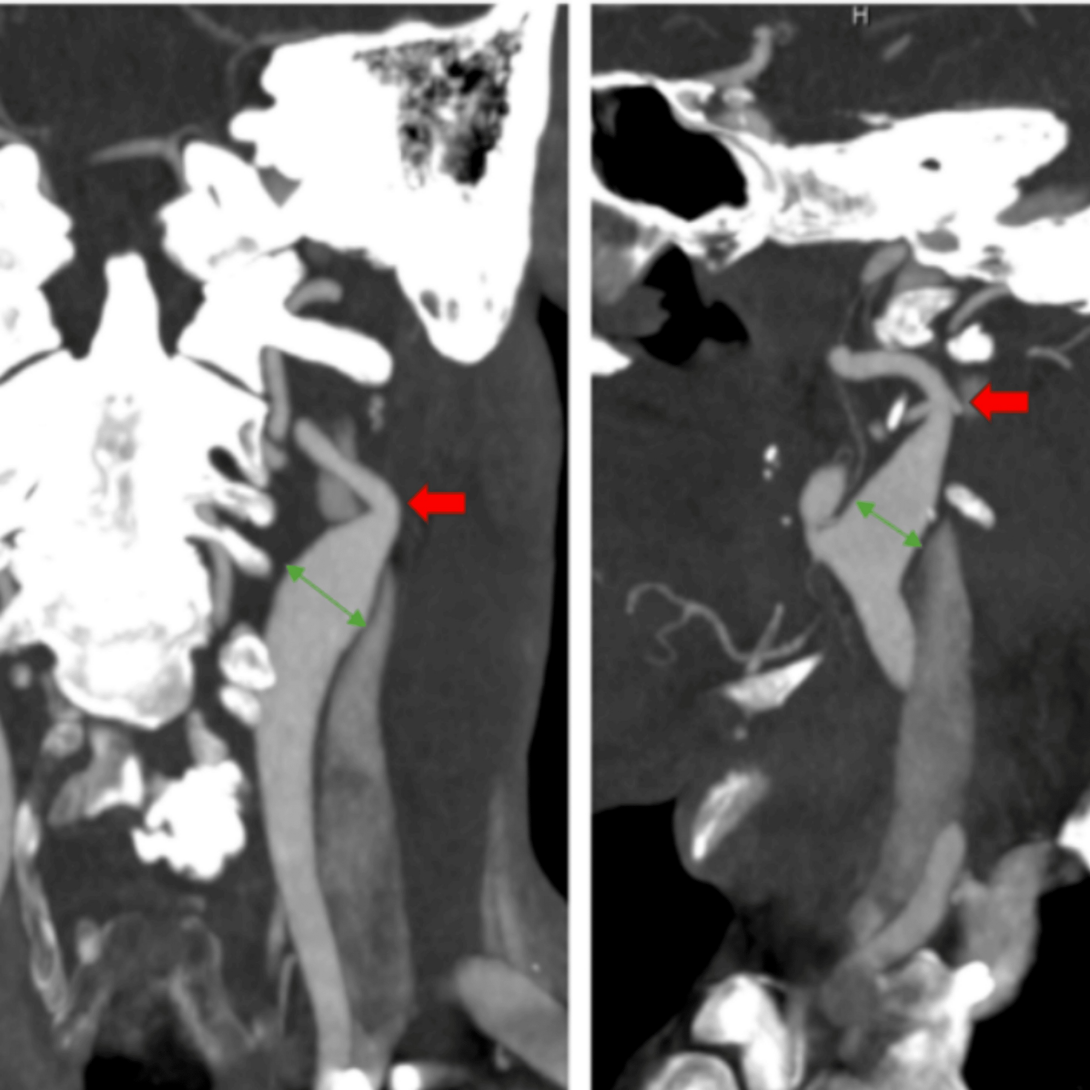
Computed tomography angiography (CTA) of the head and neck. CTA of the head and neck demonstrating tortuous carotid arteries, significant stenosis, and bilateral bulbar dilation; all features that may indicate underlying fibromuscular dysplasia. The red arrow demonstrates significant stenosis of the left carotid artery. The green arrow indicates significant carotid bulbar dilation.

After the left heart catheterization was completed, the patient’s troponin level continued to rise to greater than 27,000 ng/L over the subsequent 24 hours, and a transthoracic echocardiogram showed a new-onset cardiomyopathy with an ejection fraction of 35-39% and no significant wall motion or valvular abnormalities. To further evaluate common arterial beds associated with FMD, a carotid and renal artery duplex were obtained. The carotid ultrasound demonstrated highly tortuous bilateral internal and external carotid arteries with concerns for fibromuscular dysplasia, while the renal duplex showed normal-appearing vasculature. As per the cardiothoracic surgical consultant's request, a computed tomography angiography (CTA) aorta and CTA head and neck were obtained. While the classic "string of beads" finding was not appreciated, the CTA head and neck did demonstrate bilateral carotid tortuosity, significant bilateral non-atherosclerotic carotid stenosis, and bilateral carotid bulb dilation which are all consistent with FMD angiography (Figure 3).

As previously mentioned, the patient endorsed pulsatile tinnitus and chronic migraines, consistent with cerebrovascular FMD due to the turbulent blood flow through the tortuous carotid arteries. Thus, while in-patient, the neurology consultants evaluated the patient for chronic migraines and started him on divalproex sodium (Depakote) in addition to his home anti-seizure medication regimen due to its lack of vasoconstrictive properties. For suspected FMD-induced migraines, avoidance of vasoconstrictive migraine medications remains paramount. Upon initiation of Depakote, the patient denied recurrence of his migraines and has remained headache-free for the first time in nearly 15 years at a two-week follow-up, further substantiating a clinical phenotype consistent with FMD. The patient was instructed to continue with close neurology and cardiology follow-up and was started on carvedilol and lisinopril for afterload reduction and to prevent negative cardiac remodeling in the setting of transient cardiomyopathy. He was advised to monitor for new neurological symptoms, recurrence of chest pain, and development of syncope. The patient was instructed to close neurology and cardiology follow-ups at monthly intervals and to repeat angiographic studies if new symptoms develop. Furthermore, the patient was established with a genetic counselor for further evaluation of genetic connective tissue disorders, and the patient was instructed to limit alcohol consumption, remain free of tobacco products, and improve his dietary and exercise habits.

## Discussion

In this case, a patient was found to have a distal SCAD with an FMD-like clinical phenotype in the setting of chronic migraines, pulsatile tinnitus, and a previously unexplained TIA. As mentioned previously, fibromuscular dysplasia is an arteriopathy involving stenotic and ectatic portions of an arterial lumen [8]. As evidenced in recent literature reviews, SCAD, though an uncommon cause of acute coronary syndrome, has a significant association with FMD and other arteriopathies with clinical FMD phenotypes in any age and sex, with some estimates predicting that more than half of all SCAD patients have underlying FMD [9]. It remains important to note, however, that FMD often presents as a poly-vascular disease with more common complications including renal artery stenosis, aortic aneurysm and dissection, and carotid artery dissection. Some experts have even suggested pan-vascular scanning once an initial arterial disruption has occurred if there is evidence of multi-arterial bed injury on initial screening tests (i.e., arterial duplexes) [10]. Although the extent of vascular scanning is controversial, as it may lead to incidental findings that have no pathologic correlation, it is universally agreed that the development of SCAD warrants additional investigation into other arterial beds, as well as a thorough genetic evaluation of underlying etiology.

Given these potentially significant arterial disruptions, greater emphasis is placed on SCAD and FMD screening for primary prevention. Some reviews have noted that cardiac risk optimization, including adequate exercise, hypertension control, and lipid-lowering therapies can help prevent potentially devastating vascular sequelae [9]. However, the current American College of Cardiology recommendation is for 30-40 minutes of moderate aerobic exercise five to seven days per week, as extreme exercise increases arterial shear stress and increases the risk of SCAD in the FMD population due to high-impulse perfusion on the arterial beds [11]. Therefore, in patients with SCAD, close cardiology follow-up is required for exercise prescription to enjoy the benefits of exercise without further damaging arterial beds.

Many studies have further demonstrated the importance of conservative therapies in patients with SCAD, regardless of the underlying etiology. A recent systematic review has demonstrated that a conservative approach is the favored treatment modality in patients with SCAD [12]. Additionally, retrospective studies have shown that revascularization increases the risk of vessel occlusion and in-hospital death in SCAD patients compared to initial conservative medical management [13]. Most conservative medical therapies are patient-dependent and highly individualized based on comorbidities, including hypertension, diabetes, and tobacco usage. The majority of patients benefit from strict blood pressure control, optimization of tolerated anti-lipid therapies, beta-blockade for systolic restoration and prevention of cardiac remodeling, and the addition of either aspirin monotherapy (SAPT) or dual antiplatelet therapy (DAPT) with no significant difference between antiplatelet options [14,15]. Additionally, further investigation into genetic conditions (e.g., Ehlers-Danlos syndrome and Loeyz-Dietz syndrome) may be indicated if the patient has a characteristic phenotype for these conditions.

Lastly, post-SCAD patients oftentimes experience difficulty in recovery due to fear of recurrence and depression [16]. Studies have demonstrated that patients with SCAD experience high ranges of negative emotions and are often in need of great social support following their medical event [16]. Failure to adequately treat post-SCAD anxiety can lead to avoidance behaviors and reduced adherence to follow-up care. Furthermore, patients with SCAD often endorse feeling inadequately informed about their diagnosis, and they typically have a poor understanding of their prognosis and treatment strategies, including the role of blood pressure control and important dietary and exercise modifications. Therefore, psychosocial interventions remain critically important in the overall multi-modal management of SCAD. The conservative medical management options for SCAD are shown in Table 1.

**Table 1:** Multimodal conservative management options in spontaneous coronary artery dissection. This table describes multimodal options for the treatment of SCAD, including pharmacologic, behavioral modifications, and various psychosocial intervention strategies. SCAD: Spontaneous coronary artery dissection; SAPT: Single antiplatelet therapy; DAPT: Dual antiplatelet therapy.

Pharmacologic	Behavioral modifications	Psychosocial interventions
Anti-hypertensives (ace-inhibitors and angiotensin receptor blockers preferred)	30-40 minutes of moderate aerobic exercise 5-7 days per week	Online support groups
Lipid-lowering therapies (preferably highest intensity tolerated statin)	Cardiac rehabilitation for all patients	Allowing time to physically and mentally recover before returning to work
Beta-blockers (cardio-selective beta-blockade is preferred)	Avoidance of Valsalva, excessive straining, and high-intensity exercise in extreme conditions	Specific appointments with primary physician to discuss education and information about SCAD
Anti-platelet therapy (no significant difference between SAPT and DAPT)	No current restrictions on skydiving, scuba diving, or sexual intercourse	Family counseling and education

## Conclusions

This case report demonstrates the importance of careful investigation into the presentations of vascular diseases. Specifically, this case report demonstrates an instance of SCAD without all of the characteristic angiographic features of FMD, and details treatments including blood pressure control, risk factor modification with aspirin and high-intensity statin therapy to stabilize the arterial wall, and non-vasoconstrictive migraine therapies to decrease symptoms resulting from turbulent arterial blood flow. Additionally, the importance of mental health support is addressed, as many post-SCAD patients experience anxiety and fear of recurrence. In this way, this report highlights the multimodal conservative treatment strategies for SCAD and emphasizes the role of psychosocial therapies. More research into the optimization of diagnostic imaging criteria and molecular biomarkers is required to truly elucidate the phenotypic variability of FMD and the corresponding clinical features that are associated with its manifestation. By preventing the recurrence of arterial events and managing associated symptoms, further elucidation of this disease spectrum may help optimize individualized treatments and improve long-term health outcomes in this patient subset.
